# Effect of biodegradable chelators on induced phytoextraction of uranium- and cadmium- contaminated soil by *Zebrina pendula* Schnizl

**DOI:** 10.1038/s41598-019-56262-9

**Published:** 2019-12-24

**Authors:** Li Chen, Dan Wang, Chan Long, Zheng-xu Cui

**Affiliations:** College of Life Science and Engineering, Southwest University of Science and Technology, Mianyang, 621010 China

**Keywords:** Ecology, Environmental sciences

## Abstract

This study investigated the effect of ethylenediamine-N,N′-disuccinic acid (EDDS), oxalic acid (OA), and citric acid (CA) on phytoextraction of U- and Cd-contaminated soil by *Z. pendula*. In this study, the biomass of tested plant inhibited significantly following treatment with the high concentration (7.5 mmol·kg^−1^) EDDS treatment. Maximum U and Cd concentration in the single plant was observed with the 5 mmol·kg^−1^ CA and 7.5 mmol·kg^−1^ EDDS treatment, respectively, whereas OA treatments had the lowest U and Cd uptake. The translocation factors of U and Cd reached the maximum in the 5 mmol·kg^−1^ EDDS. The maximum bioaccumulation of U and Cd in the single plants was 1032.14 µg and 816.87 µg following treatment with 5 mmol·kg^−1^ CA treatment, which was 6.60- and 1.72-fold of the control groups, respectively. Furthermore, the resultant rank order for available U and Cd content in the soil was CA > EDDS > OA (U) and EDDS > CA > OA (Cd). These results suggested that CA could greater improve the capacity of phytoextraction using *Z. pendula* in U- and Cd- contaminated soils.

## Introduction

Uranium (U) is an important radioactive element and widely used in irradiation breeding, insect disease prevention, radiotherapy, nuclear reactor, and other industrial sectors in the form of U compounds and metallic U^1^. The development of nuclear industry as well as U mineral activities have been affecting the quality of soils in natural environments^2^. According to a survey, the average content of U in topsoil (0–20 cm) was 3.03 mg/kg in China^3^; however, the U content in the contaminated soil around U tailings is 3.21–62.37 mg·kg^−1^ in Hunan Province, China^4^. Multiple heavy metals (U, Cd, Cr, Fe, Pb, and Cu) have been found in U tailings area^5^. Meanwhile, Cd has become one of the most severe concerns in U tailings area due to its high mobility and toxicity in the soil environment^6^. Therefore, U tailing contaminated soils need be paid more attention in certain areas of China because of extensive industry activities of mining^7^. To date, the remediation technologies of metals contaminated soils, such as adsorption^8^, electrokinetic remediation^9^, soil washing^10^, and phytoextraction^11^ have been widely applied for reducing the total and/or available metals concentration in soils. Among these, phytoextraction is regarded as one of the most effective treatments because of its simple operation in practical applications. Meanwhile, it is also cost-effective and eco-friendly. However, low shoot uptake and translocation results in a low accumulation capability in plant shoot. Therefore, many methods, including chelators, have been widely applied to accumulate higher quantities of metals in plant shoot, through improving the bioavailability of the metals in soil and stimulating metal uptake in tested plant^12,13^.

In principle, chelators can be divided into two types: natural and artificial chelators. Natural chelators are mainly low-molecular-weight organic acids (LMWOA) such as, oxalic acid (OA) and citric acid (CA), which can increase the solubility and potential bioavailability of metals in soil, and OA and CA have been widely used in phytoremediation enhancement of metal-polluted soils^14–17^. Artificial chelators include diethylenetriaminepentaacetic acid (DTPA), ethyl diglycol acetate (EDGA) and ethylenediaminetetraacetic acid (EDTA), which can chelate insoluble metals to soluble species in soil^18^. Meanwhile, they usually have stronger capacity to chelate metals in soil compared with low-molecular-weight organic acids^19,20^. However, DTPA, EDGA, and EDTA have negative impacts that include low biodegradability in soil and increasing the risk of leaching of metals into groundwater^21,22^. Considering these negative effects, EDDS and CA have been widely used to increase the capacity of metals translocation from contaminated soil to harvestable parts of the tested plant because of strong chelate capacity and good biodegradability^23,24^. Lan *et al*. showed that 2 mmol·kg^−1^ EDDS treatment increased significantly shoot Cd concentrations in *Sigesbeckia orientalis* L^25^. The results of Yang *et al*. indicated that 5 mmol·kg^−1^ CA had the best effect on U phytoremediation by rye grass^26^.

*Zebrina pendula* Schnizl is a fast-growing evergreen herbage found at a uranium tailing site in Hengyang, Hunan province, China. The results of our preliminary experiment have shown that *Z. pendula* has the higher potential to absorb and accumulate U compared with other tested plants (Table S1). Meanwhile, the species also have advantages of strong tolerance to U, high biomass, and easy management.

In this study, we investigated whether the biodegradable chelators could increase the phytoextraction efficiency of U and Cd from the soil. The specific objectives of this study were to: (1) investigate the influence of three chelators on the biomass production of *Z. pendula*; (2) analyze the potential of different chelators to improve U and Cd phytoextraction; (3) assess the suitable dosage of chelators for enhancing the effect of U and Cd phytoextraction.

## Materials and Methods

### Materials

The seeds of *Z. pendula* were purchased from farm product market of Fucheng district, Mianyang City, Sichuan Province, China. All soil samples were collected from the topsoil (0–20 cm) in the Longshan vegetable garden (ferralosols), and were not contaminated by heavy metals. Soil pH was measured using a pH electrode in a 1:2.5 soil/water ratio^27^. The organic matter (OM) content was determined according to the method of Nelson and Sommers^28^ using the samples sieved through a 100 mesh (150 μm) sieve. The available nitrogen (N), phosphorus (P), and potassium (K) contents were measured according to Shen *et al*.^29^ using the samples sieved through a 100 mesh (150 μm) sieve. The basic physicochemical properties of the soil are shown in Table 1.

**Table 1:** Selected physico-chemical properties and U and Cd concentration of the soil.

Organic matter (g·kg^−1^)	pH	Cation exchange capacity (mmol·kg^−1^)	U content (mg·kg^−1^)	Cd content (mg·kg^−1^)	Available (mg·kg^−1^)		
					N	P	K
18.99	6.88	115.64	3.21	0.29	166.46	41.35	57.03

### Experimental design

The present study simulated U- and Cd-contaminated soil in a greenhouse. First, the tested soil (ferralosols) was naturally air-dried. The weeds and gravel in the soil were removed, and the soil was crushed and mixed. Subsequently, each plastic pot with a hole at the bottom was filled with 3.0 kg of grounded soil. Uranium (15 mg·kg^−1^) and Cd (15 mg·kg^−1^) were spiked into the air-dried soils by uniformly spraying an aqueous solution of UO_2_(CH_3_CO_2_)_2_·2H_2_O and CdCl_2_·2.5H_2_O onto the soil, and base fertilizer was applied in a single dose that contained (NH_4_)_2_SO_4_, KH_2_PO_4_, and K_2_SO_4_ powders (nitrogen content: 225 mg·kg^−1^, phosphorus content: 65 mg·kg^−1^, and potassium content: 227 mg·kg^−1^)^5^. Soil ageing was conducted in the pots containing the artificially contaminated soil in the greenhouse for another 35 days. The pots were arranged in a completely randomized design. The seeds of the *Z. pendula* were sowed at pollution-free farmland near the greenhouse. After 20 days of growing seedlings, three uniform healthy seedlings of the *Z. pendula* with 2–3 fronds were transplanted from the pollution-free farmland into each pot. All treatments (36 pots in total) were arranged in the greenhouse (15–25 °C) in a completely randomized design. The moisture content of soil in the pots was regularly adjusted according to Chen *et al*.^5^.

On the 90th day after the transplanting of *Z. pendula*, EDDS, OA, and CA were applied to the soils at rates of 0 (control group, CK), 2.5 (low concentration), 5 (moderate concentration) and 7.5 mmol·kg^−1^ (high concentration) in solutions, respectively. An equal amount of aqueous solution (distilled water) was used as the control group. The 100 mL solution of chelator were irrigated slowly to the soil around the roots in three batches (each batch was 33 mL in a pot, and the third batch was 34 ml) at 3-day intervals. After the chelators were applied, the soil was irrigated regularly. The tested plants were harvested after 1 week in the third batch, and measurements were taken.

### Biomass production

The plant samples were carefully removed, washed with tap water followed by distilled water. The samples were divided into shoots and roots. The samples were dried at 75 °C after 30 min at 105 °C for 48 h. The dried plant samples were weighed using an electronic balance.

### U and Cd concentration

The U and Cd concentrations of dried plant samples (shoots and roots) were measured by Chen *et al*.^5^ Concentrations of U and Cd in plant samples were determined using an inductively coupled plasma mass spectrometry (ICP-MS) (Agilent 7700x; PerkinElmer, Waltham, Massachusetts, USA).

### Available U and Cd content

The soil samples around the roots were collected, air-dried at room temperature, sifted (0.1 mm), and weighed (1.0000 g). In this study, we employed the single extraction method, which was used for available U and Cd extraction^30,31^. Mehlich III extractant (50 mL) (0.2 N CH_3_COOH, 0.25 N NH_4_NO_3_, 0.015 N NH_4_F, 0.013 N HNO_3_, and 0.001 N EDTA adjusted to pH 2.5) was mixed with each soil sample, oscillated for 30 min, and then filtered. The available U and Cd content in the soil was measured using the ICP-MS by Elrashidi *et al*.^31^.

### Computational method

1 $${\rm{TF}}={{\rm{C}}}_{{\rm{shoot}}}/{{\rm{C}}}_{{\rm{root}}}$$

2 $${\rm{Bioaccumulation}}={{\rm{C}}}_{{\rm{shoot}}{\rm{or}}{\rm{root}}}\times {{\rm{M}}}_{{\rm{shoot}}{\rm{or}}{\rm{root}}}$$

3 $${\rm{Single}}\,{\rm{metal}}\,{\rm{conc}}.=({{\rm{C}}}_{{\rm{shoot}}}\times {{\rm{M}}}_{{\rm{shoot}}}+{{\rm{C}}}_{{\rm{root}}}\times {{\rm{M}}}_{{\rm{root}}})/({{\rm{M}}}_{{\rm{shoot}}}+{{\rm{M}}}_{{\rm{root}}})$$

where the C_shoot_ and C_root_ is the concentration of heavy metals (mg·kg^−1^) in the shoot and root, respectively. The M_shoot_ and M_root_ is the mass of shoots and roots, respectively.

### Statistical analysis

All statistical tests were performed using Excel 2013, SPSS 23.0, and Origin 9.0 for Windows. Values are expressed as mean ± standard error (SE) (triplicate). Least significant difference (LSD) was applied to test for significant differences (*p* < 0.05).

## Results

### Growth parameter

**Figure 1:**
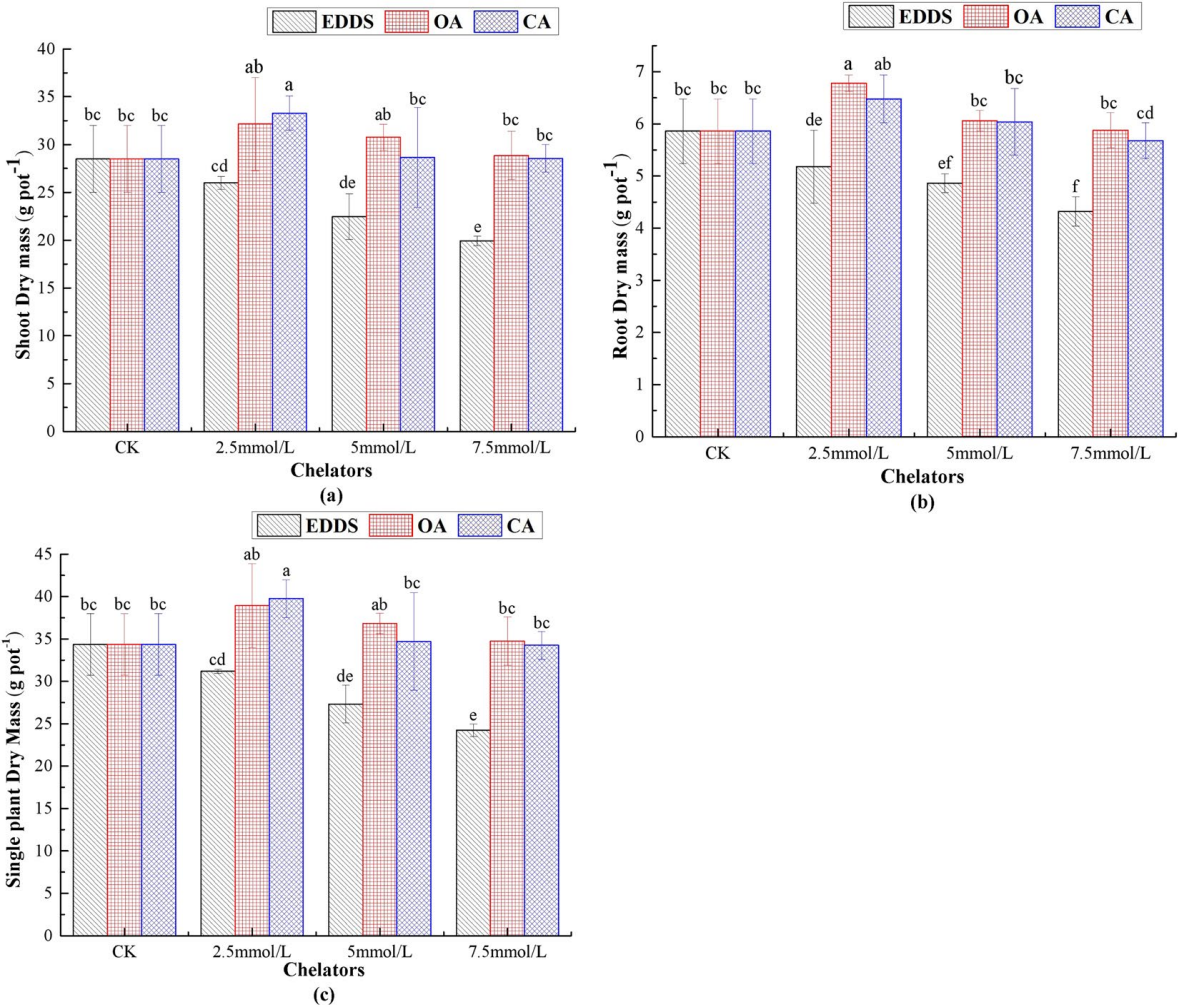
Influence of chelators application on shoot dry mass (**a**), root dry mass (**b**), and single plant dry mass (**c**) of *Z. pendula* under U and Cd stress. EDDS, OA, and CA represents different chelator application, respectively. CK indicates control. Values are reported as means ± SE. Means with different letter are significantly different (*p* < 0.05) using LSD test.

Figure 1 displays the variation in dry weight of shoots, roots, and single plants of *Z. pendula* in different treatments. The dry mass of shoots showed average increases of 12.7% and 16.77% in the 2.5 mmol·kg^−1^ OA and CA treatments, respectively, and with increasing concentrations, the shoot dry mass decreased constantly. Compared with the control, the plant growth was severely inhibited, and the shoot dry mass decreased 30.04% following treated with 7.5 mmol·kg^−1^ EDDS (Fig. 1(a)). The variation trends in the root dry mass under the different concentrations of chelators were the same as that of the shoot dry mass. The root dry mass increased 15.7% and 10.58% in 2.5 mmol·kg^−1^ OA and CA treatments compared with the control, respectively (Fig. 1b). However, the root dry mass was 4.32 g in 7.5 mmol·kg^−1^ EDDS treatment, which was 26.28% lower than that of the control (5.86 g) (Fig. 1(b)). The effect of the chelators on the single plant mass was different (Fig. 1(c); CA > OA > EDDS). Furthermore, 2.5 mmol·kg^−1^ OA and 2.5 mmol·kg^−1^ CA treatments promoted the growth of *Z. pendula*, but the biomass production decreased with increasing the concentration. This could be attributed to the fact that low concentrations of small molecular organic acids alleviate the toxicity of heavy metals to plants, whereas high concentrations cause two-fold increase in toxicity of the chelators and heavy metals to plant physiological growth^32,33^.

### U uptake and translocation

The potential capacity of EDDS, OA, and CA to remove U from contaminated soil is illustrated in Table 2. Compared with the control, the U absorbtion of different treatments increased by 12.41% to 444.25%. The order of U absorbtion was as follows: CA > EDDS > OA. Meanwhile, CA significantly promoted U absorption by *Z. pendula*. with the single plant U concentration increasing by 444.25% in the 5 mmol·kg^−1^ treatment, reaching a maximum of 30.03 mg·kg^−1^. Meanwhile, the U concentration in the shoots and roots also reached a maximum in the 5 mmol·kg^−1^ CA treatment (9.04 mg·kg^−1^ and 127.62 mg·kg^−1^, respectively), which was 7.35-fold and 6.1-fold higher than that of the control group (1.23 mg·kg^−1^ for U and 20.91 mg·kg^−1^ for Cd), respectively (Table 2). The TF of U of *Z. pendula* increased in each treatment. Compared with the control, the TF of U increased by 15.25% to 77.97%, and the promotion rank order was: EDDS > CA > OA (Table 2). Meanwhile, the maximum TF of U was observed following treated with 5 mmol·kg^−1^ EDDS, which was higher than that of the other treatments.

**Table 2:** Impact of chelators application on U uptake and translocation of *Z. pendula*.

Treatments (mmol·kg^−1^)	Shoot U concentration (mg·kg^−1^)	Root U concentration (mg·kg^−1^)	Single U concentration (mg·kg^−1^)	Translocation factor (TF)
Control	1.23 ± 0.22i	20.91 ± 2.86e	4.59 ± 0.56e	0.059
EDDS 2.5	2.76 ± 0.35 f	34.08 ± 4.75c	7.92 ± 0.67de	0.081
EDDS 5.0	5.96 ± 0.23c	57.67 ± 6.72b	15.19 ± 1.06c	0.105
EDDS 7.5	4.66 ± 0.47d	54.93 ± 3.51b	13.63 ± 1.06c	0.086
OA 2.5	1.62 ± 0.31hi	23.18 ± 3.38de	5.16 ± 0.43e	0.079
OA 5.0	1.96 ± 0.25gh	27.29 ± 1.8cde	6.18 ± 0.42e	0.074
OA 7.5	2.34 ± 0.32fg	32.32 ± 3.46 cd	7.40 ± 0.48de	0.073
CA 2.5	3.95 ± 0.18e	49.40 ± 6.79b	11.36 ± 0.92 cd	0.082
CA 5.0	9.04 ± 0.39a	127.62 ± 8.11a	30.03 ± 3.19a	0.072
CA 7.5	8.01 ± 0.51b	118.67 ± 9.95a	26.40 ± 1.07b	0.068

Note: data are mean ± SE (n = 3). One-way ANOVA were performed for each parameter. Different letters within the same parameter indicate significant differences (*p* < 0.05) according to the LSD test. Single U concertation is content of U in one (single) plant calculated by the Eq. 3.

### Cd uptake and translocation

The chelators promoted the absorption of Cd by *Z. pendula* in U- and Cd- contaminated soil (Table 3). The order of Cd absorption was EDDS > CA > OA. The Cd concentration in the roots and single plant reached a maximum in the 7.5 mmol·kg^−1^ EDDS treatment (148.48 mg·kg^−1^ and 32.09 mg·kg^−1^, respectively), which was 1.99-fold and 2.32-fold that of the control, respectively. However, the shoot Cd content reached a maximum of 5.34 mg·kg^−1^ at the 5 mmol·kg^−1^, which increased 4.32-fold compared to the control. This may be due to the ability that the plant translocated heavy metal from roots to shoots was inhibited in high concentration EDDS treatments. The TF of Cd reached the maximum in 5 mmol·kg^−1^ EDDS, which increased 109.38% compared to that of the control (Table 3). However, the TF of Cd decreased following treatment with OA and CA. These results indicates that it is more feasible to apply EDDS to improve the efficiency of Cd phytoextraction than OA and CA treatments.

**Table 3:** Impact of chelators application on Cd uptake and translocation of *Z. pendula*.

Treatments (mmol·kg^−1^)	Shoot Cd concentration (mg·kg^−1^)	Root Cd concentration (mg·kg^−1^)	Single Cd concentration (mg·kg^−1^)	Translocation factor
Control	1.38 ± 0.16e	74.51 ± 12.17d	13.84 ± 1.95d	1.28 × 10^−3^
EDDS 2.5	2.57 ± 0.3 cd	107.63 ± 9.61bc	19.95 ± 3.45c	1.11 × 10^−3^
EDDS 5.0	7.34 ± 0.99a	116.53 ± 11.96b	26.70 ± 3.75b	2.70 × 10^−3^
EDDS 7.5	6.11 ± 1.19b	148.48 ± 17.14a	32.09 ± 2.7a	1.38 × 10^−3^
OA 2.5	1.40 ± 0.21e	86.47 ± 6.18d	16.20 ± 1.65 cd	0.93 × 10^−3^
OA 5.0	1.61 ± 0.2e	90.85 ± 8.91 cd	16.23 ± 1.35 cd	0.99 × 10^−3^
OA 7.5	1.72 ± 0.21de	88.24 ± 7.87d	16.34 ± 1.05 cd	1.08 × 10^−3^
CA 2.5	1.63 ± 0.18e	90.69 ± 9.67 cd	16.07 ± 1.95 cd	0.99 × 10^−3^
CA 5.0	3.08 ± 0.16c	120.13 ± 9.17b	23.48 ± 3.9bc	1.09 × 10^−3^
CA 7.5	3.18 ± 0.31c	125.67 ± 17.85b	23.56 ± 3.3bc	1.01 × 10^−3^

Note: data are mean ± SE (n = 3). One-way ANOVA were performed for each parameter. Different letters within the same parameter indicate significant differences (*p* < 0.05) according to the LSD test. Single Cd concertation is content of Cd in one (single) plant calculated by the Eq. 3.

### U and Cd accumulation

**Figure 2:**
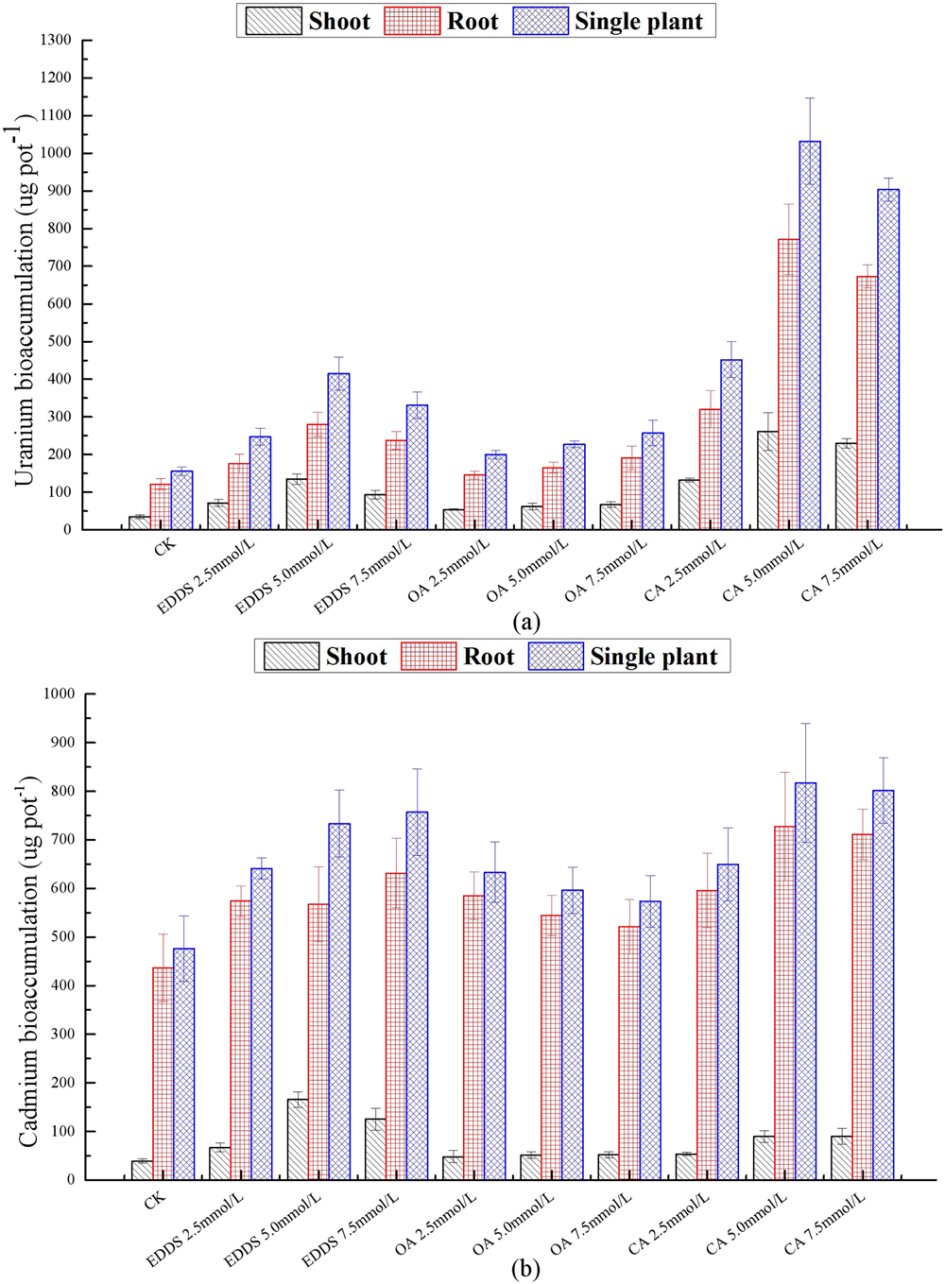
Effects of chelators application on U (**a**) and Cd (**b**) bioaccumulation (shoot, root, and single plant) in *Z. pendula*. The error bar represents the standard error of triplicate.

Heavy metal accumulation in plants is a key index for evaluating remediation efficiency, and the overground accumulation is more important than the underground accumulation. The ability of EDDS, OA, and CA to alter the accumulation of U (a) and Cd (b) are illustrated in Fig. 2. Each treatment promoted the U accumulation by the plants (Fig. 2a). The order of U accumulation was as follows: CA > EDDS > OA. The U accumulation in the shoots, roots, and single plants increased significantly in the CA treatments. The maximum U accumulations in the shoots, roots, and single plants was 130.36, 385.71 and 516.07 µg in the 5 mmol·kg^−1^ CA treatment, respectively, which was significantly (*p* < 0.05) higher than that of the control and other treatments (Fig. 2a). Furthermore, CA is an inexpensive, natural, small molecule organic acid that does not produce secondary pollution to the environment; therefore, it is feasible to apply CA to remove the U from U- and Cd-contaminated soil. As shown in Fig. 2(b), the ability of different treatments to assist *Z. pendula* in accumulating Cd was inconsistent. EDDS has a more significant effect on shoot Cd accumulation compared with the other treatments. The shoot Cd accumulation reached a maximum (165.63 µg) in 7.5 mg·kg^−1^ EDDS, which increased 3.21-fold compared to the control (Fig. 2b). However, the maximum of root and single plant Cd accumulation was 727.47 µg and 816.87 µg in 5 mg·kg^−1^ CA treatment, respectively. These results show that EDDS has better capacity to promote Cd accumulation in shoot than OA and CA, whereas CA has a more significant effect on the root Cd accumulation compared to EDDS and OA.

### Available U and Cd content in soils

The addition of the EDDS and CA increased the available U and Cd content in the soil (Table 4). The order of U activation by the chelators was CA > EDDS > OA. In the CA treatment, the available U content first increased and then decreased, reaching a maximum (14.58 mg·kg^−1^) at 5 mmol·kg^−1^, which was significantly (*p* < 0.05) higher than that of the control and the other treatments. The available U content in the CA treatment was 1.86-fold and 1.42-fold higher than that of the control and 5 mmol·kg^−1^ EDDS treatment, respectively (Table 3). However, the ability of EDDS to activate Cd in the soil was better than OA and CA treatments. The maximum available Cd content was 8.93 mg·kg^−1^ in 5.0 mmol·kg^−1^ EDDS treatment, which was 43.33% higher than that of the control (6.23 mg·kg^−1^) (Table 3). In general, CA could more efficiently activate U from the soil, whereas EDDS has greater effect on activating Cd from the soil. The reason was that the ability to activate U and Cd and chelation stability constants were not identical among three chelators. Furthermore, this difference could be attributed to the variation in soil type, soil physicochemical properties, etc.

**Table 4:** Effects of chelators application on available U and Cd content in the soil (mg·kg^−1^).

Treatments	Concentration (mmol·kg^−1^)	Available U content (mg·kg^−1^)	Available Cd content (mg·kg^−1^)
Control	0	7.81 ± 1.09d	6.23 ± 0.54bc
EDDS	2.5	8.29 ± 0.66 cd	8.33 ± 0.68a
EDDS	5.0	10.21 ± 1.13bc	8.93 ± 0.87a
EDDS	7.5	10.65 ± 1.24b	8.23 ± 0.76a
OA	2.5	8.08 ± 0.79d	6.02 ± 0.61c
OA	5.0	8.14 ± 0.71d	6.24 ± 0.56bc
OA	7.5	8.25 ± 0.4d	6.32 ± 0.53bc
CA	2.5	10.26 ± 0.99b	6.39 ± 0.56bc
CA	5.0	14.58 ± 1.99a	7.14 ± 0.83b
CA	7.5	13.96 ± 1.91a	6.67 ± 0.69bc

Note: data are mean ± SE (n = 3). One-way ANOVA were performed for each parameter. Different letters within the same parameter indicate significant differences (*p* < 0.05) according to the LSD test.

### Correlation analysis

The correlation coefficients among plant growth, uptake of metals, translocation factor (TF) of metals, available metal content in the soil, and accumulation of metal by the plant are shown in Table 5 (U) and Table 6 (Cd). The shoot U concentration, root concentration, and available U content were extremely significantly positive correlated with U accumulation in the plant (*p* < 0.01). Meanwhile, there was no obvious correlation between plant growth (shoot and root dry mass) and U accumulation. This result indicates that the U concentration of the shoot and root and available U content in the soil were the main ingredients in the phytoextraction process compared with plant growth. For Cd, the shoot Cd concentration was extremely significantly positive correlated with shoot Cd accumulation (*p* < 0.01). Dramatically, there was no obvious correlation between shoot Cd concentration and Cd accumulation in single plant, possibly due to that Cd is mainly accumulated in the root of *Z. pendula*. In addition, the Cd concentration of the shoots (roots) exhibited a significantly negative correlation with the dry mass of the shoots (roots), suggesting that high concentration Cd in the tested plant might engender an adverse effect on the plant growth.

**Table 5:** Correlation coefficient among plant growth, U uptake, translocation factor (TF) of U, available U content in the soil, and U accumulation (n = 10) (Significant at *p < 0.05 and extremely significant **p < 0.01).

	Shoot dry mass	Root dry mass	Shoot U concentration	Root U concentration	Available U content	TF
Shoot dry mass	1					
Root dry mass	0.952**	1				
Shoot U concentration	−0.261	−0.240	1			
Root U concentration	−0.115	−0.102	0.971**	1		
Available U content	−0.131	−0.116	0.952**	0.990**	1	
TF	−0.585	−0.542	0.183	−0.034	0.023	1
Shoot U accumulation	0.029	−0.021	0.971**	0.974**	0.978**	0.019
Root U accumulation	0.036	0.048	0.936**	0.982**	0.973**	−0.136
Single U accumulation	0.020	0.030	0.948**	0.979**	0.977**	−0.097

**Table 6:** Correlation coefficient among plant growth, Cd uptake, translocation factor (TF) of Cd, available Cd content in the soil, and Cd accumulation (n = 10) (Significant at *p < 0.05 and extremely significant **p < 0.01).

	Shoot dry mass	Root dry mass	Shoot Cd concentration	Root Cd concentration	Available Cd content	TF
Shoot dry mass	1					
Root dry mass	0.952**	1				
Shoot Cd concentration	−0.870**	−0.822**	1			
Root Cd concentration	−0.732*	−0.718*	0.769*	1		
Available Cd content	−0.855**	−0.851**	0.862**	0.680*	1	
TF	−0.809**	−0.750*	0.973**	0.610	0.836**	1
Shoot Cd accumulation	−0.797**	−0.746*	0.986**	0.769**	0.838**	0.967**
Root Cd accumulation	−0.085	−0.069	0.291	0.715*	0.203	0.146
Single Cd accumulation	−0.365	−0.333	0.599	0.855**	0.474	0.477

## Discussion

The application of chelators in phytoremediation can chelate metals in soil and facilitate absorption by tested plants^34,35^. However, chelator treatments of high dose also have a toxic effect on tested plant growth, resulting in even death of the plants^36^. In this study, the results indicate that CA and OA treatments promote the growth of *Z. pendula* at low concentrations (2.5 mmol·kg^−1^). It might be due to that the growth of the tested plant was not negatively affected by low dose heavy metal concentrations, whilst the biomass yield was increased following treatment with moderate CA and OA concentrations^36,37^. Nevertheless, all chelators in the present study inhibited the growth of *Z. pendula* following treatment with high concentration, and EDDS treatment had stronger inhibition effect on the growth of *Z. pendula* compared with OA and CA treatments. This is agree with the conclusions of Moslehi *et al*.^38^ that treating *Helianthus annuus* L. with 5 mmol·kg^−1^ EDDS decreased significantly the dry weight of shoot and root in uncontaminated soils. Chen *et al*.^39^ found that the dry mass of the shoot and the root decreased in 7.5 mmol·kg^−1^ EDDS treatment in the Co contaminated soils. The reason is possible that low concentrations of organic acids can mitigate the toxicity of heavy metals in plants^40^. However, excessive concentration of chelators lead to a significant increase in metal ions concentration in the soil solution, which in turn causes more severe stress to plants. In addition, excessive concentration of chelators have a certain degree of toxicity to plant^41^; therefore, the tested plant biomass decreased^42^. Wei *et al*. reported that low concentrations (2.5 mmol·kg^−1^) of L glutamic acid N, N-diacetic acid (GLDA) increased the biomass of *Sedum alfredii*, whereas high concentrations (10 mmol·kg^−1^) produced toxic effects on growth, which was consistent with the findings of the present study^43^.

Chelators can promote absorption, translocation, and accumulation of heavy metals in plants, possibly due to that chelators can enhance desorption of metals from the soil matrix to the soil solution, chelate insoluble metals to water soluble species in soil, change the form of heavy metals in soil, increase the content of available heavy metals in soil, and facilitate metal transport into the xylem and increase metal translocation from roots to shoots^19,44^. In the present study, the addition of chelators increased the available U and Cd content in the soil. CA and EDDS had the best effect on the absorption of U and Cd by *Z. pendula*, respectively. These results indicate that the available U and Cd content in the soil was directly correlated to the absorption of U and Cd by *Z. pendula*. Meanwhile, correlation analysis also showed that the available U and Cd content in the soil was extremely significant positive with the concentration of U and Cd in *Z. pendula*. Furthermore, chelators have different chelation effects on different heavy metals in soil due to mutual selectivity. For example, Zhang *et al*.^45^ indicated that EDTA was the most effective of the three chelators for Pb phytoremediation due to the promotion of the available Pb content in the soil.

In the current study, the effects of EDDS, OA, and CA treatments on the uptake and translocation of U and Cd from contaminated soil are different. The chelators significantly promoted the U and Cd uptake, and the order of U and Cd uptake was CA > EDDS > OA (U), EDDS > CA> OA (Cd), respectively. In this study, the concentrations of U and Cd in the shoots and roots of *Z. pendula* increased highest by 6.35- and 6.1-fold for CA treatment and 4.32- and 0.99-fold for EDDS treatments, respectively, when compared with those of the control groups. The effects on phytoextraction in heavy metals contaminated soil are different using different chelators, which may be because the ability that chelators changed the form of heavy metals in the soil is different due to its differences of physicochemical property. Zhang *et al*. reported that applying EDDS treatments to Cd and Pb phytoextraction significantly (*p* < 0.05) increased the Cd concentration of the shoots and roots than the CA treatments; meanwhile, the available Cd content in EDDS treatments is also higher than the CA treatments^45^. Hu *et al*. tested CA-assisted U phytoremediation on *Macleaya cordata* and indicated that the U concentration of the shoots and roots in the 10 mmol·kg^−1^ CA treatment was significantly higher than (*p* < 0.05) the EDDS and OA treatments^46^. In addition, the TF is one of most important indicators that determine whether a plant has the potential to remove metals from the contaminated soils. The TFs of U and Cd of *Z. pendula* were much lower than 1 in control groups, indicating that U and Cd was mainly absorbed by the roots of *Z. pendula*. However, the maximum TFs of of U and Cd were 1.78- and 2.11-fold higher than those in the control groups, respectively, which was consistent with the observation by Wan *et al*.^47^ and Zhang *et al*.^45^.

The accumulation of metals in the tested plant is the key index to evaluate the phytoextraction. The chelator treatments effectively promoted the accumulation of U and Cd by *Z. pendula*, and the effect of different treatments on accumulation was also different. This study indicates that applying CA caused higher U concentrations and accumulation amount in the shoots and the roots compared with other chelators. This finding is consistent with the result of Jagetiya *et al*.^36^ that the chelator strengthened U accumulation in Indian mustard follow the order of CA > EDTA > OA > NTA. The U accumulations of the shoots and single plants following treatment with 5 mmol·kg^−1^ CA were 6.48-fold and 5.6-fold higher than those in the control groups, respectively. Yang *et al*. also showed that the application of 5 mmol·kg^−1^ CA increased the U accumulation coefficient in the shoots and roots of rye grass by 2.31-fold and 1.67-fold that of the control, respectively^26^. The maximum of shoot Cd accumulation was 165.63 µg in 7.5 mg·kg^−1^ EDDS treatment. Nevertheless, the maximum of root and single plant Cd accumulation was 727.47 µg and 816.87 µg in 5 mg·kg^−1^ CA treatment, respectively. The main reason is that biomass production of the tested plant was inhibited significantly in higher dosages of EDDS treatments than the CA treatments.

The application of chelators can increase the bioavailability of metals in soil-plant system through chelation with metals^48^. The results showed that the available U and Cd content in the soil was influenced by the three chelators, and the order of metals activation was CA > EDDS > OA (U) and EDDS > CA > OA (Cd), respectively. This reason possible is due to the mutual selectivity of the metals and the various chelating agents. This is consistent with the results of Lozano *et al*., who showed that particular chelator is suitable for particular metal in soil^49^, such as CA treatment was more efficient to increase the solubility of uranium than EDDS treatment^50^.

High dosages of chelators result in greater toxicity in plants, as indicated by such signs as leaf etiolation and wilting^51^. Similar results were observed in the present study. Heavy metals damage the cytoplasmic membrane and normal biochemical mechanisms for controlling transport in plants. Chelators can be used to transfer metals from soil to plants by forming metal chelates. This results in the excessive accumulation of metals in the tested plant tissues, which has a toxic effect on plant growth and can even cause plant dehydration and death^36^. Due to the high concentration of metals in the current study, further investigation is required to determine whether the toxicity of the chelators is direct or indirect. Based on these results, when conducting chelation-induced phytoextraction, it is important to remember that excessive chelators treatments may engender adverse effects on the growth and development of the plant species.

## Conclusion

This study showed that applications of the chelators significantly enhanced phytoextraction of U and Cd by *Z. pendula*. in the contaminated soils. High concentration (7.5 mmol·kg^−1^) of EDDS treatment significantly (*p* < 0.05) inhibited the plant growth, but low (2.5 mmol·kg^−1^) concentrations of CA and OA were significantly (*p* < 0.05) conducive to the growth of the tested plant. Chelators enhanced the uptake, translocation, and accumulation of Cd and U by *Z. pendula*. The maximum U and Cd accumulation amount were observed with the 5 mmol·kg^−1^ CA treatment, and were significantly higher (*p* < 0.05) than that of the control. Meanwhile, the available U and Cd concentrations in the soil were influenced by all tested chelator treatments, and the order of available metal contents in the soil was as follow: CA > EDDS > OA (U) and EDDS > CA > OA (Cd), respectively. Available U content in the soil is one of the main parameters controlling accumulation of U in the *Z. pendula* through correlation analysis. In U- and Cd-contaminated soil, CA was effective in improving absorption and accumulation amounts of U and Cd by *Z. pendula*. These findings would be beneficial to increase removal efficiency of U and Cd from contaminated soils. Furthermore, the potential impacts on soil properties after soil remediation will be further considered in future studies.

## Supplementary information

Supplementary information

Dataset 1
