# Supplementary material for: Effect of biodegradable chelators on induced phytoextraction of uranium- and cadmium- contaminated soil by Zebrina pendula Schnizl
Source: Sci Rep. 2019 Dec 24;9:19817. doi: 10.1038/s41598-019-56262-9 (PMC6930220; doi:10.1038/s41598-019-56262-9)
Supplement: Supplementary file 1 — Supplementary information [file 41598_2019_56262_MOESM1_ESM.pdf]

Article Title: Effect of biodegradable chelators on induced phytoextraction of uranium- and cadmium- contaminated soil by *Zebrina pendula* Schnizl

First name: Li
Family name: Chen
Affiliations: Southwest University of Science and Technology
Address: Qing long road, Mianyang, Sicchuan, P.R. China
Email: chenlixkd@163.com

First name: Dan
Family name: Wang
Affiliations: Southwest University of Science and Technology
Address: Qinglong road, Mianyang, Sicchuan, P.R. China
Email: wangdanxkd@163.com

First name: Chan
Family name: Long
Affiliations: Southwest University of Science and Technology
Address: Qinglong road, Mianyang, Sicchuan, P.R. China
Email: 976365503@qq.com

First name: Zheng-xu
Family name: Cui
Affiliations: Southwest University of Science and Technology
Address: Qinglong road, Mianyang, Sicchuan, P.R. China
Email: 2570836197@qq.com

Table S1. U concentration of shoots and roots and translocation factor (TF) under U contaminated soils

Plant species	Shoot U concentration	Root U concentration	TF
Setaria viridis (L.) Beauv.	1.33＋0.16cd	26.78＋4.41c	0.050
Artemisia argyi Levl. et Van	0.56＋0.10fgh	16.15＋1.02de	0.035
Bidens pilosa L.	2.01＋0.20b	9.96＋1.23fgh	0.203
Ipomoea aquatica Forsk	0.47＋0.08ghi	6.52＋0.75h	0.073
Chrysanthemum coronarium L.	0.76＋0.10efg	28.6＋3.15c	0.027
Brassica juncea var. multiceps	3.41＋0.54a	8.46＋1.00gh	0.403
Alternanthera philoxeroides (Mart.) Griseb.	0.39＋0.05hi	1.43＋0.09i	0.273
Commelina communis	1.38＋0.06c	13.47＋0.92ef	0.103
Linum usitatissimum L.	0.39＋0.06hi	7.23＋0.38gh	0.055
Tagetes patula L.	1.58＋0.15c	16.3＋0.92de	0.097
Aloe vera (Haw.) Berg	0.74＋0.06efg	45.51＋4.67b	0.016
Erigeron canadensis L	1.03＋0.17de	19.07＋1.49d	0.054
Abelmoschus esculentus (Linn.)Moench	0.28＋0.06hi	11.84＋2.08efg	0.023
Eleusine indica (L.) Gaertn.	0.58＋0.1fgh	14.04＋2.03ef	0.041
Abutilon theophrasti Medicus	0.82＋0.06ef	19.04＋1.98d	0.043
Zebrina pendula Schnizl	3.32＋0.50a	52.26＋7.21a	0.065
Amaranthus tricolor L.	0.75＋0.09efg	30.94＋3.92c	0.025
Helianthus annuus L.	0.19＋0.03i	15.22＋1.86de	0.013

Note: data are mean ± SE (n = 3). One-way ANOVA were performed for each parameter. Different letters within the same parameter indicate significant differences ($p < 0.05$) according to the LSD test.
